# Metabolic profiles of male meat eaters, fish eaters, vegetarians, and vegans from the EPIC-Oxford cohort^1^,^2^

**DOI:** 10.3945/ajcn.115.111989

**Published:** 2015-10-28

**Authors:** Julie A Schmidt, Sabina Rinaldi, Pietro Ferrari, Marion Carayol, David Achaintre, Augustin Scalbert, Amanda J Cross, Marc J Gunter, Georgina K Fensom, Paul N Appleby, Timothy J Key, Ruth C Travis

**Affiliations:** 3 Cancer Epidemiology Unit, Nuffield Department of Population Health, University of Oxford, Oxford, United Kingdom;; 4 International Agency for Research on Cancer, Lyon, France; and; 5 Department of Epidemiology and Biostatistics, School of Public Health, Imperial College London, London, United Kingdom

**Keywords:** EPIC-Oxford, mass spectrometry, metabolomics, vegan, vegetarian

## Abstract

**Background:** Human metabolism is influenced by dietary factors and lifestyle, environmental, and genetic factors; thus, men who exclude some or all animal products from their diet might have different metabolic profiles than meat eaters.

**Objective:** We aimed to investigate differences in concentrations of 118 circulating metabolites, including acylcarnitines, amino acids, biogenic amines, glycerophospholipids, hexose, and sphingolipids related to lipid, protein, and carbohydrate metabolism between male meat eaters, fish eaters, vegetarians, and vegans from the Oxford arm of the European Prospective Investigation into Cancer and Nutrition.

**Design:** In this cross-sectional study, concentrations of metabolites were measured by mass spectrometry in plasma from 379 men categorized according to their diet group. Differences in mean metabolite concentrations across diet groups were tested by using ANOVA, and a false discovery rate–controlling procedure was used to account for multiple testing. Principal component analysis was used to investigate patterns in metabolic profiles.

**Results:** Concentrations of 79% of metabolites differed significantly by diet group. In the vast majority of these cases, vegans had the lowest concentration, whereas meat eaters most often had the highest concentrations of the acylcarnitines, glycerophospholipids, and sphingolipids, and fish eaters or vegetarians most often had the highest concentrations of the amino acids and a biogenic amine. A clear separation between patterns in the metabolic profiles of the 4 diet groups was seen, with vegans being noticeably different from the other groups because of lower concentrations of some glycerophospholipids and sphingolipids.

**Conclusions:** Metabolic profiles in plasma could effectively differentiate between men from different habitual diet groups, especially vegan men compared with men who consume animal products. The difference in metabolic profiles was mainly explained by the lower concentrations of glycerophospholipids and sphingolipids in vegans.

## INTRODUCTION

Human metabolism is influenced by dietary, lifestyle, environmental and genetic factors (1, 2), and individuals with different dietary habits might therefore have different metabolic profiles in blood. Metabolomics is the identification and quantification of metabolites (i.e., low-molecular-weight reactants, intermediates, or products of biochemical reactions) in a biological system (1, 3). In recent years, metabolomics has been introduced into epidemiologic research with the aims of identifying novel risk factors for disease and biomarkers of diet and improving our understanding of disease mechanisms (4).

To date, only one study has been published on metabolic profiles in individuals from different habitual diet groups with respect to intake of food from animal sources, e.g., meat eaters and vegetarians. This study found a clear separation between omnivores and lactovegetarians (5).

We aimed to investigate differences in concentrations of individual circulating metabolites and in metabolic profiles between male meat eaters, fish eaters, vegetarians, and vegans from the Oxford component of the European Prospective Investigation into Cancer and Nutrition (EPIC-Oxford)^6^ by using a targeted metabolomic approach. The metabolites investigated were acylcarnitines, amino acids, biogenic amines, glycerophospholipids, hexose, and sphingolipids related to lipid, protein, and glucose metabolism. The current study will add to this existing literature by investigating the metabolic profile in plasma of 4 distinct habitual diet groups.

## METHODS

### Study population

From 1993 to 2000, 65,000 men and women ≥20 y of age were recruited from across the United Kingdom into the EPIC-Oxford cohort (6). This cohort aims to investigate associations between diet, lifestyle, and cancer risk in individuals with different long-term dietary habits; thus, a large number of vegetarians and vegans were recruited. Participants were mostly recruited via post (89%) or via their general practitioner’s surgery. All participants gave written informed consent, and the protocol for EPIC-Oxford was approved by a multicenter research ethics committee (MREC/02/0/90).

At recruitment, participants completed a validated semiquantitative food-frequency questionnaire (FFQ) (7, 8) with additional questions on lifestyle, body size, and previous disease. Participants were also asked to have a blood sample taken at their local general practitioner’s surgery; participants were not required to fast, and time since last food or drink was recorded. Blood samples were sent at ambient temperature to the laboratory, where they were processed for long-term storage in liquid nitrogen (−196°C) until 2011 and subsequently in electric freezers (−80°C). Time between blood collection and processing was also recorded.

For the current cross-sectional analysis, the eligibility criteria were as follows: male sex, age 30–49 y, provision of a blood sample at recruitment, known smoking status and diet group, response to ≥80% of the relevant questions in the FFQ and a daily energy intake between 3.3 and 16.7 MJ (800–4000 kcal), and no prior cancer (excluding nonmelanoma skin cancer), cardiovascular disease, or treatment of any long-term illness or condition at recruitment. A total of 110 vegans (who do not eat meat, fish, dairy products, or eggs) were eligible, and all were between the ages of 30 and 39 y; 4 in every 5 vegans (randomly selected) between the ages of 40 and 49 y were selected. In addition, eligible meat eaters, fish eaters (who do not eat meat but do eat fish), and vegetarians (who eat neither meat nor fish) were randomly selected in equal numbers within the age strata. Thereby, 98 men from each diet group were available for this study.

### Laboratory analysis

Plasma samples were assayed by tandem mass spectrometry at the International Agency for Research on Cancer, Lyon, France, with the use of the targeted metabolomic assay BIOCRATES Absolute*IDQ* p180 Kit. Amino acids (*n* = 21) and biogenic amines (*n* = 9) were separated by liquid chromatography before injection into the mass spectrometer, whereas flow injection analysis was used for acylcarnitines (*n* = 18), glycerophospholipids (*n* = 82), hexose (*n* = 1), and sphingolipids (*n* = 14). A total of 145 metabolites were quantified. Before this assay, samples had gone through 2 or 3 thaw-freeze cycles; samples from each diet group were equally distributed by the number of cycles.

The diet groups were randomly distributed between analytic batches. In each batch, 4–6 blinded quality-control samples from pooled plasma were included (26 in total). The median CV of all quality-control samples combined (SD divided by the mean) was 6.9% for acylcarnitines, 11.8% for amino acids, 9.8% for biogenic amines, 7.8% for glycerophospholipids, 5.9% for hexose, and 7.7% for sphingolipids.

Metabolites with CVs >20% (5 metabolites), with >10% of the measurements outside the measurable range (19 metabolites) or with missing metabolite information for >5% of participants (4 metabolites), were excluded from the analyses. A total of 27 metabolites were excluded. Furthermore, men with missing information on any of the included metabolites (13 men) were excluded. These exclusions left 118 metabolites (9 acylcarnitines, 19 amino acids, 3 biogenic amines, 72 glycerophospholipids, hexose, and 14 sphingolipids) and 379 men (95 meat eaters, 97 fish eaters, 91 vegetarians, and 96 vegans) for further analyses.

For 13 of the included metabolites, some men (<10%) had measurements outside the measurable range. Measurements below the limit of detection were set to half the lowest measured concentrations (ranging from 0.015 to 7.753 μmol/L; applicable to 7 acylcarnitines for 1 to 15 men). Measurements below the limit of quantification (0.1 μmol/L for serotonin only, applicable to 10 men) were set to half the limit of quantification. Finally, measurements above the highest concentration calibration standards (400 μmol/L for leucine, histidine, threonine, and ornithine and 800 μmol/L for glutamate; applicable to 1 to 2 men) were set to the highest standards.

The nomenclature of the metabolites was published previously (9). In brief, fatty acid side chains were labeled “Cx:y,” where x and y denote the number of carbon atoms and double bonds, respectively. Acylcarnitines were abbreviated according to the fatty acid side chain. All glycerophospholipids were phosphatidylcholines, and subclasses were separated by the number of fatty acids side chains and type of bond. “LysoPC a” denotes phosphatidylcholines with one fatty acid side chain bound with an acyl bond, “PC aa” denotes 2 acyl side chains, and “PC ae” denotes one acyl and one alkyl side chain. Sphingolipids were sphingomyelins with a hydroxyl group [SM(OH)] or without a hydroxy group attached and were also labeled according to the fatty acid side chain. Both phosphatidylcholines and sphingomyelins are abundant phospholipids in cell membranes. Hexose is the sum of a range of monosaccharides with 6 carbon atoms, including glucose, fructose, and galactose. Amino acids and biogenic amines were labelled by using their full name.

### Diet and body size

Participants were asked whether they ate meat, fish, dairy products, or eggs, and they were categorized as meat eaters, fish eaters, vegetarians, and vegans. The FFQ had questions on 130 foods and drinks, 113 of which were relevant to vegetarians and vegans. Mean daily intakes were estimated by using specified portion sizes (10), and mean daily nutrient intakes were estimated mostly by using the fifth edition of *McCance and Widdowson’s The Composition of Foods* and its supplements (11–20).

Height and weight were used to calculate BMI (in kg/m^2^). In addition to self-reported measurement, height and weight were also measured in a subsample of the cohort. Self-report and measured values showed good agreement (*r* > 0.9) (21). Measured BMI was used if available.

### Statistical analysis

Participant and blood sample characteristics were compared across diet groups by using the Kruskal-Wallis 1-factor ANOVA, and a chi-square test was used to test for differences between the diet groups for continuous and categorical variables, respectively (except for hemolysis, for which Fisher's exact test was used).

All metabolite concentrations were log transformed to approximate the normal distribution. Associations of metabolite concentrations with participant and blood sample characteristics (besides diet group) were investigated by ANOVA adjusted for age (30–39, 40–44, or 45–49 y) and BMI (<22.5, 22.5–24.9, ≥25, or unknown). To account for multiple comparisons in this analysis and other analyses of individual metabolite concentrations, a false discovery rate–controlling procedure (Benjamini-Hochberg) was used. First, the *P* values were sorted and ranked from the lowest p(1) to the highest p(m). Adjusted *P* values were then calculated by using (*i*/m) × α, where *i* is the rank of the original *P* value and α = 0.05. The values were sequentially compared, and the null hypothesis was rejected for the *i* tests with *P* values < (*i*/*m*) × α.

Differences in geometric mean concentrations of individual metabolites across diet groups were tested by using ANOVA adjusted for personal and blood sample characteristics, i.e., age (categorized as above), BMI (categorized as above), smoking status (never, former, or current), alcohol intake (<1, 1–7, 8–15, or ≥16 g/d), time since last food or drink at blood collection (<1.5 h, 1.5–2.9 h, 3.0–4.4 h, ≥4.5 h, or unknown), and time between blood collection and processing (fourths of the distribution corresponding to <25 h, 25–41 h, 41–72 h, ≥72 h, or unknown). Further adjustments for energy intake, time of day at blood collection, hemolysis and lipemic status did not materially change the results; these results are thus not shown.

Spearman’s rank correlation coefficients were calculated between pairs of metabolites. Principal component analysis based on the covariance matrix was conducted on log-transformed metabolite concentrations (22). The choice of numbers of components to retain was based both on a visual examination of the scree plot and on the percentage of variation explained; we aimed to capture ∼60% of the total variation.

Score plots of the retained components were plotted for visual examination of separation between diet groups (23), and ANOVA was used to test for differences in the diet group mean scores. To assess the potential effect of confounding, the ANOVA was adjusted for potential confounders (as defined for the analysis of individual metabolites). Loading plots, in which the points represent the metabolites, were examined to assess which metabolites might explain any separation between diet groups. The orientation of the loading plot matches that of the score plot; thus, in combination, these 2 plots can be used to identify metabolites responsible for differences in diet group scores (23).

To assess how much of the total variability in the metabolomics data was explained solely by diet group and not by participant or blood sample characteristics (as defined above), the principal component partial *R*^2^ method was used (24). In brief, principal component analysis was performed on the metabolite concentrations, and multiple linear regression of the retained principal components against covariates (here diet group, and participant or blood sample characteristic) were fitted. The *R*_partial_^2^ statistic was then determined for all covariates for each principal component. Finally, the overall *R*_partial_^2^ statistic was calculated for each covariate as a weighted average.

## RESULTS

### Participant and blood sample characteristics

BMI varied by diet group. The highest BMI was observed in meat eaters, followed by fish eaters, vegetarians, and vegans (**Table 1**). The intake of nutrients also differed by diet group. Meat eaters had the highest energy intake, followed by vegetarians, fish eaters, and vegans. The intake of energy from protein was also highest in meat eaters, followed by fish eaters, vegetarians, and vegans. The opposite was seen for carbohydrate intake; vegans had the highest intake, followed by vegetarians, fish eaters, and meat eaters. Overall, intake of energy from fat did not vary by diet group, but differences were observed for subtypes of fat. The intake of energy from SFAs was highest in meat eaters, followed by vegetarians, fish eaters, and vegans. For MUFAs, meat eaters also had the highest intake, followed by fish eaters, vegans, and vegetarians. The intake of energy from PUFAs was higher the more that animal products were excluded from the diet. The intake of alcohol was highest in fish eaters, followed by vegetarians, meat eaters, and vegans. Of the factors related to blood collection, an association was seen only for time between blood collection and processing, with the process delay being shorter in meat eaters than in the other groups.

**TABLE 1:** Characteristics and nutrient intakes of 379 men in EPIC-Oxford by diet group^1^

	Meat eaters (*n* = 95)	Fish eaters (*n* = 97)	Vegetarians (*n* = 91)	Vegans (*n* = 96)	*P*^2^
Participant characteristics					
Age at blood collection, y	44 (37, 44)^3^	40 (36, 45)	43 (36, 44)	40 (35, 44)	0.9
BMI^4^, kg/m^2^	24.4 (22.4, 26.0)	22.7 (21.1, 24.2)	22.7 (21.8, 25.1)	22.1 (20.5, 23.8)	0.0001
Current smoker, *n* (%)	14 (14.7)	9 (9.3)	6 (6.6)	7 (7.3)	0.1
Very physically active,^4,5^ *n* (%)	57 (60.0)	60 (61.9)	61 (67.0)	65 (67.7)	0.3
Nutrient intake					
Energy, kJ	9198 (7997, 11,045)	8751 (7518, 10,127)	9,012 (7597, 10,971)	7652 (6084, 8866)	0.0001
Protein, % of energy	14.98 (13.61, 16.82)	13.64 (12.29, 15.42)	13.26 (11.86, 14.14)	12.64 (11.68, 13.90)	0.0001
Carbohydrates, % of energy	51.30 (47.19, 55.56)	52.14 (48.32, 57.42)	54.28 (48.56, 58.49)	55.60 (52.27, 60.56)	0.0001
Fat, % of energy	31.96 (28.61, 35.00)	32.19 (27.22, 35.02)	31.46 (27.43, 35.39)	30.40 (25.39, 34.30)	0.2
SFA, % of energy	11.18 (9.37, 13.15)	10.38 (8.31, 12.42)	10.47 (8.58, 12.60)	6.21 (5.12, 7.66)	0.0001
MUFA, % of energy	10.85 (9.45, 12.22)	10.19 (8.63, 11.47)	9.86 (8.64, 11.38)	10.07 (7.90, 11.79)	0.02
PUFA, % of energy	6.15 (4.77, 7.65)	6.68 (5.52, 8.38)	7.27 (5.43, 8.68)	9.71 (7.63, 11.98)	0.0001
Alcohol, g/d	2.68 (0.87, 5.40)	3.40 (1.11, 6.88)	3.35 (1.34, 7.66)	1.92 (0.35, 6.04)	0.04
Blood sample characteristics					
Medication or supplement taken,^4^ *n* (%)	57 (60.0)	60 (61.9)	61 (67.0)	65 (67.7)	0.3
Time since last food or drink,^4^ h	2.0 (1.0, 3.5)	2.3 (1.3, 4.5)	2.5 (1.5, 4.5)	2.5 (1.3, 4.2)	0.05
Time of day of collection,^4^ h:min	11:50 (10:10, 15:27)	10:45 (09:28, 15:30)	10:30 (09:40, 15:05)	10:40 (09:45, 16:04)	0.3
Time from collection to processing,^4^ h	25.8 (23.4, 67.1)	44.1 (25.8, 71.9)	43.7 (24.6, 71.4)	41.9 (24.7, 72.3)	0.006
Lipemic, *n* (%)	23 (24.2)	13 (13.4)	23 (25.3)	17 (17.7)	0.1
Hemolysis, *n* (%)	1 (1.1)	6 (6.2)	6 (6.6)	3 (3.1)	0.2

1 EPIC-Oxford, Oxford component of the European Prospective Investigation into Cancer and Nutrition.

2 Differences between diet groups were tested by using the Kruskal-Wallis one-factor ANOVA and chi-square test for continuous and categorical variables, respectively (except for hemolysis, for which Fisher's exact test was used).

3 Median; IQR in parentheses (all such values).

4 Information was missing for some participants: BMI, *n* = 20; physical activity level, *n* = 2; medication or supplement taken, *n* = 2; time since last food or drink, *n* = 11; time of day of blood collection, *n* = 10; and time from collection to processing, *n* = 10.

5 Defined according to a modified version of the Cambridge Physical Activity Index (25).

### Associations between metabolites and covariates

Concentrations of histidine, serine, creatinine, and 12 glycerophospholipids varied according to age group after allowance for multiple testing (**Supplemental Table 1**). BMI, energy intake, and alcohol intake were each related to concentrations of several glycerophospholipids (BMI, *n* = 26; energy, *n* = 26; alcohol, *n* = 26), a few sphingolipids (BMI, *n* = 3; energy, *n* = 6; alcohol, *n* = 6), certain amino acids (BMI, *n* = 5; energy, *n* = 1; alcohol, *n* = 1), and an acylcarnitine. In addition, BMI and energy intake were associated with kynurenine concentration, and BMI was associated with concentrations of creatinine and hexose. Time since last food or drink and time of day at blood collection were associated with some amino acids (time since last food or drink, *n* = 5; time of day at blood collection, *n* = 5) and one biogenic amine (kynurenine). Time of day at blood collection was also associated with one glycerophospholipid. The time between blood collection and processing was associated with some acylcarnitines (*n* = 4) and amino acids (*n* = 16) plus a few glycerophospholipids (*n* = 7) and hexose. Lipemic samples had higher concentrations of several amino acids (*n* = 3), glycerophospholipids (*n* = 23), and sphingolipids (*n* = 2) than did nonlipemic samples, whereas hemolysis was related to some amino acids (*n* = 6), one glycerophospholipid, hexose, and 2 sphingolipids. Smoking status, level of physical activity, and use of medication or supplements on the day of blood collection were not associated with metabolite concentrations after allowance for multiple testing.

### Analysis of individual metabolite concentrations

**FIGURE 1:**
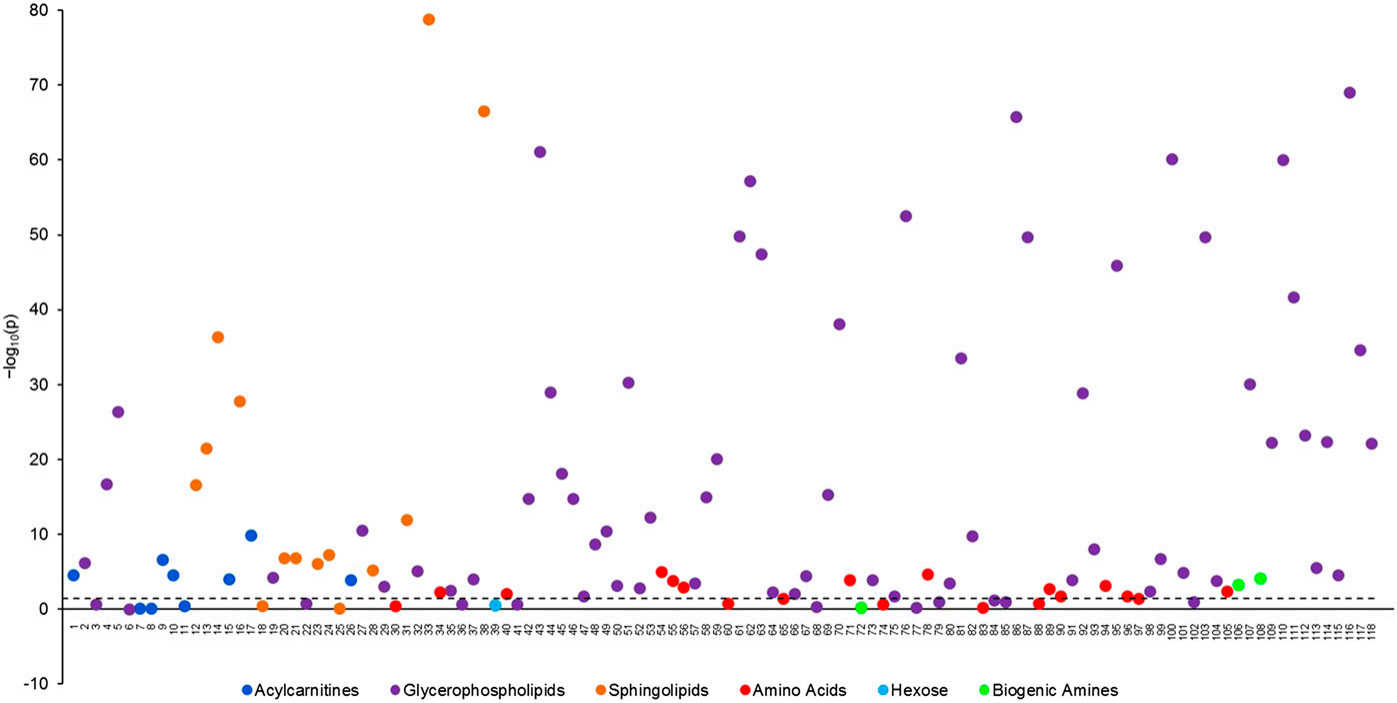
Statistical significance of the associations between diet group and metabolite concentrations plotted as –log_10_ (*P* values) in 379 men from the Oxford component of the European Prospective Investigation into Cancer and Nutrition study. The dashed line shows the largest adjusted *P* value after the false discovery rate method for multiple comparisons was used, such that the null hypothesis was rejected for all *P* values lower than this (*P* = 0.0351 corresponding to –log_10_(p) =1.45). The *P* values were derived from an ANOVA comparing geometric mean metabolite concentration across the 4 diet groups, adjusted for age (30–39, 40–44, or 45–49 y), BMI (in kg/m^2^; <22.5, 22.5–24.9, ≥25, or unknown), smoking status (never, former, or current), alcohol intake (<1, 1–7, 8–15, or ≥16 g/d), time since last food or drink at blood collection (<1.5 h, 1.5–2.9 h, 3–4.4 h, ≥4.5 h, or unknown), and time between blood collection and processing (fourths of the distribution corresponding to <25 h, 25–41 h, 41–72 h, ≥72 h, or unknown). The metabolites were ordered according to a dendrogram that was created by using a dissimilarity matrix containing the values |1–ρ|, where ρ was the Spearman’s rank correlation coefficient. The complete linkage clustering method was used to determine the distance between sets of metabolites. The geometric mean and 95% CIs of metabolite concentrations by diet group are shown in Supplemental Table 2.

Overall, mean concentrations of 93 of 118 metabolites (78.8%) differed significantly by diet group after allowance for multiple testing (**Figure 1**, **Supplemental Table 2**; see **Supplemental Table 3** for full details of the metabolites, including biochemical name and class). The strongest and the highest proportions of differences were seen for the sphingolipids (85.7% differed) and glycerophospholipids (84.7% differed).

Six of 9 acylcarnitines varied by diet group; C-0, C-4, and C-5 concentrations were highest in meat eaters, followed by fish eaters, vegetarians, and vegans (Supplemental Table 2). Similarly, C-3 and C-16 were highest in meat eaters and lowest in vegans. In contrast, vegans had the highest concentration of 18:2, followed by fish eaters, vegetarians, and meat eaters. This difference in 18:2 (concentrations that were 45% higher in vegans than in meat eaters) represents the largest difference between vegans and meat eaters for those metabolites that were highest in vegans.

Twelve of 19 amino acids varied by diet group. Concentrations of leucine, valine, lysine, methionine, tryptophan, and tyrosine were generally highest in fish eaters and vegetarians, followed by meat eaters and vegans, whereas the citrulline concentration was higher in vegetarians and vegans than in meat eaters or fish eaters. The concentrations of glycine and ornithine were highest in vegans. The alanine concentration was lower in meat eaters than in the other 3 diet groups, whereas the glutamate concentration was highest in fish eaters, followed by vegetarians, and meat eaters. Glutamine was lowest in the fish eaters. The biogenic amine kynurenine was highest in vegetarians, followed by fish eaters, meat eaters, and vegans, whereas the creatinine concentration was highest in meat eaters, followed by fish eaters, vegetarians, vegans.

Of the glycerophospholipids, 61 of 72 differed across diet groups; overall, vegans tended to have the lowest concentrations. For 23 glycerophospholipids, meat eaters had the highest concentrations, followed by fish eaters, vegetarians, and vegans. This pattern was found for PC aa 36:6, and the difference of 64% between meat eaters and vegans was the largest difference observed among all the metabolites. For 5 glycerophospholipids, the lowest concentration was observed in vegans, whereas concentrations were similarly higher in the 3 other diet groups. The remaining 33 glycerophospholipids showed different patterns; the concentrations of 28 of these were also lower in vegans than in meat eaters, whereas the remaining 5 were higher in vegans than in meat eaters.

The concentrations of 12 of 14 sphingolipids differed across diet groups. For all except 3, meat eaters had the highest concentrations followed by fish eaters, vegetarians, and vegans. Similar patterns were seen for 2 additional sphingolipids (sphingomyelin 20:2 and sphingomyelin 24:1), although concentrations were similar in fish eaters and vegetarians for the former and were similar in vegetarians and vegans for the latter. For SM(OH) 24:1, the mean concentration was similar in meat eaters, fish eaters, and vegetarians, whereas vegans had the lowest concentration. No difference in hexose was seen by diet group.

Because the results of analyses of metabolite concentrations across the 4 diet groups indicated that metabolite concentrations in vegans, in particular, may differ from those in the other diet groups, we conducted additional analyses to compare *1*) metabolite concentrations in vegans compared with the other 3 diet groups combined and *2*) metabolite concentrations across the 3 nonvegan diet groups (**Supplemental Tables 4** and **5**, respectively). Mean concentrations of 86 of 118 metabolites (72.9%) differed significantly between vegans and nonvegans (Supplemental Table 4), and, as in the analysis across all 4 diet groups, the strongest and the highest proportions of the differences were seen for the sphingolipids (85.7% differed) and glycerophospholipids (79.1% differed). Fewer differences were observed between the 3 nonvegan diet groups; concentrations of only 39 of 118 metabolites (33.1%) differed significantly between meat eaters, fish eaters and vegetarians, The majority of these metabolites were glycerophospholipids and sphingolipids (29 glycerophospholipids, 6 sphingolipids, 3 amino acids, and 1 biogenic amine), and the most commonly observed pattern was for meat eaters to have the highest concentrations, followed by fish eaters and vegetarians. However, compared with meat eaters, fish eaters and vegetarians had higher concentrations of alanine and glutamine and lower concentrations of creatinine. The citrulline concentration was higher in vegetarians than in meat eaters and fish eaters.

Overall, the metabolites were highly intercorrelated; 49.7% of the correlations were significant after allowance for multiple testing (*r* ranged from −0.57 to −0.23 and 0.23 to 0.97) (**Supplemental Figure 1**). The strongest positive correlations were seen within metabolite classes, especially within the glycerophospholipids and sphingolipids. Metabolites from these 2 classes were also strongly positively correlated. Only a few negative correlations were observed—mainly for hexose and arginine but also for some acylcarnitines and amino acids.

### Analysis of metabolic profiles

Four principal components were retained for further inspection, and together they explained 60.1% of the total variation. The first principal component explained 37.7% of the total variation and was characterized by positive loadings on some glycerophospholipids (especially PC aa C32:1, PC aa C36:5, PC aa C36:6, and PC ae C34:0) and on a few sphingolipids [especially SM(OH) 14:1]. Principal component 2 explained 10.6% of the total variance, and it was primarily characterized by positive loadings on 3 acylcarnitines (C-16, 18:1, and C18:2) and negative loadings on arginine and hexose. The third principal component explained 6.3% of the total variance and was characterized by positive loadings on some glycerophospholipids (especially PC aa 32:1, PC aa C34:4, and PC aa C40:5) and negative loadings on a few sphingolipids [especially SM(OH) 16:1]. Finally, principal component 4 explained 5.5% of the total variation and was mainly characterized by positive loadings on some glycerophospholipids (especially PC ae C44:6).

**FIGURE 2:**
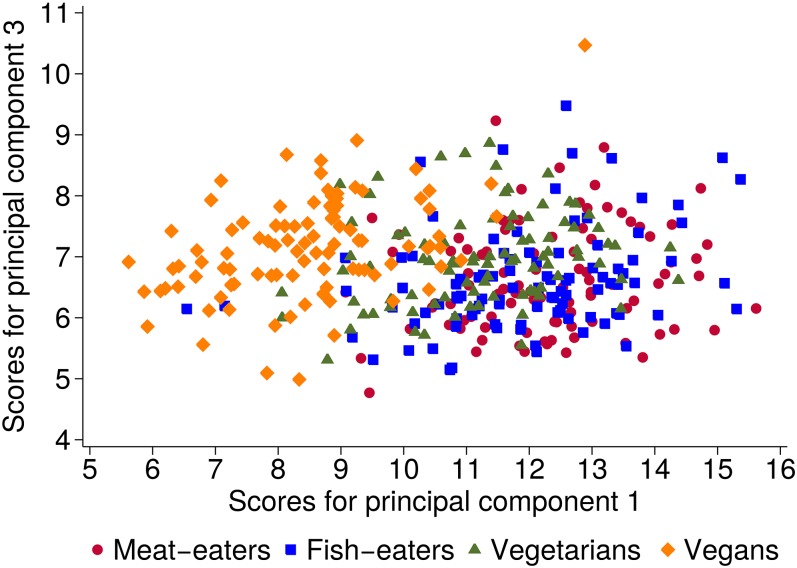
Score plot for principal components 1 and 3 by diet group. Each point represents a participant. The principal component analysis was based on the covariance matrix conducted on log-transformed metabolite concentrations (22).

The score plot of principal component 1 against 3 showed the best separation between diet groups (**Figure 2**); the separation was mainly seen on principal component 1. The comparison of mean scores showed significant differences across the diet groups for all 4 principal components (**Table 2**). For principal component 1, meat eaters had the highest score followed by fish eaters, vegetarians, and vegans, who had the lowest score (31% lower than that of meat eaters). In contrast, for principal components 2, 3, and 4, the mean score was generally highest in vegans and lowest in meat eaters. Adjustment for potential confounders did not materially change the results for principal component 1, 3, and 4, whereas differences across diet groups in scores for principal component 2 were attenuated and no longer significant.

**TABLE 2:** Principal component score by diet group in EPIC-Oxford^1^

	Mean principal component score				
Principal component	Meat eaters(*n* = 95)	Fish eaters(*n* = 97)	Vegetarians(*n* = 91)	Vegans(*n* = 96)	*P*^2^
1					
Unadjusted	12.3 (12.0, 12.6)^3^	12.0 (11.7, 12.2)	11.2 (10.9, 11.5)	8.5 (8.2, 8.8)	4 × 10^−61^
Mean change,^4^ %	100	−3	−8	−31	
Adjusted^5^	12.3 (12.0, 12.6)	11.9 (11.6, 12.2)	11.2 (10.9, 11.5)	8.6 (8.3, 8.9)	8 × 10^−52^
Mean change,^4^ %	100	−3	−9	−30	
2					
Unadjusted	3.0 (2.8, 3.2)	3.5 (3.2, 3.7)	3.4 (3.1, 3.6)	3.4 (3.1, 3.6)	0.01
Mean change,^4^ %	100	+7	+23	+20	
Adjusted^5^	3.1 (2.9, 3.3)	3.4 (3.2, 3.6)	3.3 (3.1, 3.5)	3.3 (3.2, 3.5)	0.4
Mean change,^4^ %	100	+8	+6	+7	
3					
Unadjusted	6.6 (6.5, 6.8)	6.6 (6.5, 6.8)	7.0 (6.8, 7.1)	7.2 (7.0, 7.3)	2 × 10^−6^
Mean change,^4^ %	100	0	+5	+8	
Adjusted^5^	6.5 (6.3, 6.7)	6.6 (6.5, 6.8)	7.0 (6.8, 7.1)	7.3 (7.1, 7.4)	5 × 10^−10^
Mean change,^4^ %	100	+2	+7	+12	
4					
Unadjusted	7.8 (7.7, 8.0)	7.6 (7.4, 7.7)	7.9 (7.7, 8.0)	8.4 (8.2, 8.5)	1 × 10^−11^
Mean change,^4^ %	100	−3	+1	+7	
Adjusted^5^	7.8 (7.7, 8.0)	7.6 (7.4, 7.7)	7.9 (7.7, 8.0)	8.3 (8.2, 8.5)	8 × 10^−10^
Mean change,^4^ %	100	−3	0	+6	

1 Principal component scores were derived by using principal component analysis based on the covariance matrix of log-transformed metabolite concentrations. EPIC-Oxford, Oxford component of the European Prospective Investigation into Cancer and Nutrition.

2 *P* values refer to test for difference in component score across the 4 diet groups calculated using analysis of variance.

3 Mean; 95% CI in parentheses (all such values).

4 Compared with meat eaters.

5 Adjusted for age (30–34, 35–39, 40–44, or 45–49 y), BMI (in kg/m^2^; <22.5, 22.5–24.9, ≥25, or unknown), smoking status (never, former, or current), alcohol intake (<1, 1–7, 8–15, or ≥16 g/d), time since last food or drink at blood collection (<1.5 h, 1.5–2.9 h, 3–4.4 h, ≥4.5 h, or unknown), and time between blood collection and processing (fourths of the distribution corresponding to <25 h, 25–41 h, 41–72 h, or ≥72 h, or unknown).

**FIGURE 3:**
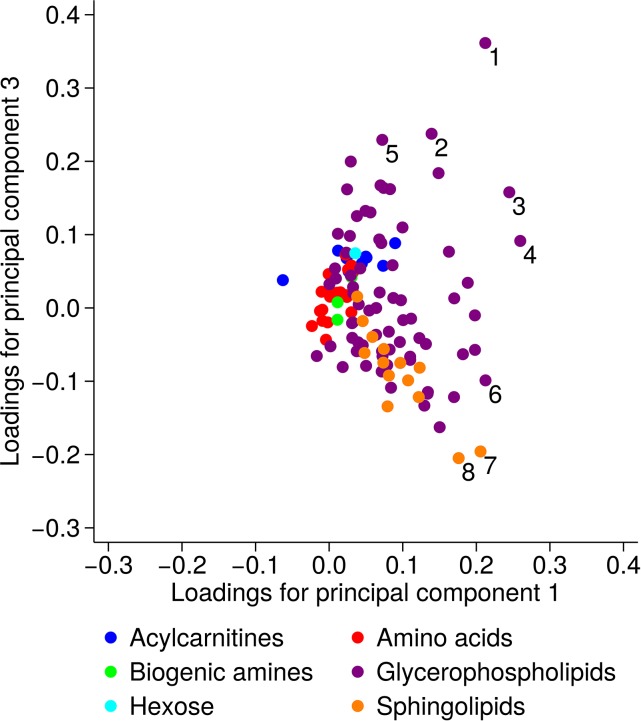
Loading plot for principal components 1 and 3. Each point represents a metabolite, and the marked metabolites are as follows: 1 PC aa 32:1, 2 PC aa 34:4, 3 PC aa 36:5, 4 PC aa 36:6, 5 PC aa 40:5, 6 PC ae 34:0, 7 SM(OH) 14:1, and 8 SM(OH) 16:1. The principal component analysis was based on the covariance matrix conducted on log-transformed metabolite concentrations (22). PC aa, phosphatidylcholine with 2 acyl side chains; PC ae, phosphatidylcholine with one acyl and one alkyl side chain; SM(OH), sphingomyelin with a hydroxy group.

The loading plot of principal components 1 and 3 showed that the separation between the diet groups—especially the vegans from the other groups—was primarily explained by lower concentrations of some glycerophospholipids and 2 sphingolipids, especially PC ae 34:0, SM(OH) 14:1, and SM(OH) 16:1 but also PC aa C32:1, PC aa C34:4, PC aa C36:5, and PC aa C36:6 (**Figure 3**).

Diet group, and personal and blood sample characteristic (i.e., age, BMI, smoking status, alcohol intake, time since last food or drink at blood collection, and time between blood collection and processing) combined explained 29.4% of the total variability in the metabolomics data. The major contributor to the variation was diet group, explaining 18.5% of the total variability, whereas the other variables each explained between 0.5% (smoking status) and 4.4% (time between blood collection and processing). Of note, time since last food or drink at blood collection only explained 1.8% of the total variation in metabolite concentrations.

## DISCUSSION

In this analysis of plasma metabolites by habitual diet group, significant differences in metabolic profiles were observed between meat eaters, fish eaters, vegetarians, and especially vegans. This difference was primarily driven by lower concentrations of some glycerophospholipids and sphingolipids in vegans than in the other diet groups, although there were also differences in levels of other metabolites; concentrations of 12 sphingolipids (86%), 61 glycerophospholipids (79%), 6 acylcarnitines (67%), 2 biogenic amines (67%), and 12 amino acids (63%) varied across the 4 diet groups, whereas no association was seen for hexose. For the vast majority of these metabolites, vegans had the lowest concentrations and the meat eaters most often had the highest concentrations.

Our findings of lower concentrations of glycerophospholipids and sphingolipids in vegans than in the other diet groups and the strong correlations between sphingolipids and glycerophospholipids are consistent with synthesis pathways and dietary sources of these metabolites. Both phosphatidylcholine and sphingomyelin (i.e., the type of glycerophospholipids and sphingolipids in this study) are provided by the diet (mainly from animal products such as eggs, poultry, and red meat) and by de novo synthesis (26, 27). Of the 2 main pathways for de novo synthesis of phosphatidylcholines, one requires choline (28) [mainly found in animal products but also in plant foods such as whole grains (27)] and the other involves methylation of phosphatidylethanolamine, which is inhibited when methionine availability is low (28). Sphingomyelins are synthesized from phosphatidylcholines (29).

Only one previous study has investigated metabolic profiles by habitual diet group, i.e., groups defined by intake of animal products; it found a clear separation between omnivores and lactovegetarians in urinary metabolic profiles (5). Of the 10 metabolites that explained the differences between male omnivores and lactovegetarians, only glycine, phenylalanine, and glucose (as part of hexose) were also part of the current study, and differences in these metabolites did not explain the differences in metabolic profiles between the diet groups in our study. However, the glycine concentration did vary by diet group in the current analysis of individual metabolites and, in line with the previous study (5), meat eaters had lower glycine concentrations than did the other diet groups. Additional studies support an association between habitual dietary intake and the metabolite concentrations. Metabolites measured in serum by using untargeted mass spectrometry were associated with intakes of several foods, including citrus fruit, meat, fish, butter, peanuts, coffee, and alcoholic drinks, and the Healthy Eating Index (30), whereas metabolite concentrations measured in urine by using ^1^H nuclear magnetic resonance (NMR) were associated with intakes of vegetable or red meat and with dietary patterns (31).

Our findings of higher circulating concentrations of creatinine and 5 of the 9 acylcarnitines in meat eaters than in vegans are broadly consistent with the findings from feeding studies that aimed to identify biomarkers of meat intake. Such studies have found higher concentrations of creatinine (biogenic amine) and acylcarnitines in urine by using a nonmetabolomics assay or NMR, after experimental diets high in meat compared with a vegetarian diet (the experimental diets contained up to 420 g meat/d compared with a mean intake of 73 g/d in meat eaters in the current study) (32, 33).

Comparisons of results from the previous studies and ours are not straightforward because of different populations, biological samples, and analytic platforms. The previous study of omnivores and lactovegetarians was conducted in a Chinese population consisting of military personnel (omnivores) and members of a Buddhist College and temple (lactovegetarians). These men likely had diets, lifestyles, and genetic make-ups very different from those of the British men in the current study and, thus, different metabolic profiles (2, 34). Moreover, different samples (urine vs. plasma) and assay methods (untargeted NMR vs. targeted mass spectrometry) were used in the previous studies.

The strengths of this study were the well-characterized cohort and the investigation of 4 distinct diet groups, the importance of which was highlighted by the marked separation of metabolic profile in vegans from that of the other diet groups. Moreover, the availability of information on a large number of covariates enabled us to investigate the independent associations between diet group and metabolite concentrations.

Pre-analytic factors such as fasting status, processing delay, and freeze-cycles could affect the concentrations of metabolites and, thus, the study results. However, time since last food or drink at blood collection and time from blood collection to processing contributed only a minor extent to the total variability in the metabolite data (explaining 1.8% and 4.4%, respectively) and thus had at most a small effect on our results. The limited effect of fasting status on the study results is in line with previous findings in EPIC (24, 35) and may be partly due to the relatively narrow time range since the last meal, i.e., most (72%) of the samples were collected within 4 h of the last food or drink. In addition, methodologic work, with use of the same assay used in the current study, has shown that most metabolites (72% and 93%) are stable after noncentrifuged blood samples are left at room temperature for 24 h and after 2 freeze-thaw cycles, respectively (36). To further limit the effect of pre-analytic factors, fasting status and process delay were controlled for in the statistical analyses, whereas the number of freeze-thaw cycles was independent of diet group in the current study.

As for other metabolomics studies, multiple testing—thereby increased risk of finding statistically significant associations purely by chance—is a limitation of the current analysis. To minimize this problem, we controlled the false discovery rate using the Benjamini and Hochberg method. Another limitation was the moderate number of participants, although it is the largest study to date to investigate metabolite profiles in individuals from different habitual diet groups. Regardless of this limitation and the application of a relatively conservative correction for multiple testing (37), many strong associations were seen between metabolites and diet group, which indicates that individuals who exclude more or less animal products from their diet truly have different metabolic profiles.

Investigating whether the observed differences in metabolic profiles by diet group may translate into differences in disease risk is beyond the scope of the current study. However, the differences in phospholipid concentrations are particularly noteworthy given the essential role of PCs in lipoprotein synthesis (28) and the previous findings from the EPIC-Oxford cohort of both a more favorable lipid profile and a lower risk of ischemic heart disease in vegetarians and vegans than in the other diet groups (38, 39). Findings of associations between prediagnostic plasma concentrations of specific phosphatidylcholines and sphingomyelins and risk of cardiovascular disease also suggest a potential role of phospholipids in the development of cardiovascular disease (40, 41).

In conclusion, metabolic profiles in plasma could effectively differentiate between men from different habitual diet groups, especially vegan men compared with men who consume animal products. The difference in metabolic profile observed was mainly explained by the vegans having lower concentrations of phospholipids in cell membranes, namely glycerophospholipids and sphingolipids. Additional research might help elucidate whether the observed differences in metabolic profiles can help explain the lower risk of some noncommunicable diseases (e.g., ischemic heart disease) in individuals who exclude some or all animal products from their diet.

## Footnotes

6 Abbreviations used: EPIC, European Prospective Investigation into Cancer and Nutrition; FFQ, food-frequency questionnaire; NMR, ^1^H nuclear magnetic resonance; SM(OH), sphingomyelin with a hydroxy group.
